# R21/Matrix-M malaria vaccine drives diverse immune responses in pre-exposed adults: insights from a phase IIb controlled human malaria infection trial

**DOI:** 10.3389/fimmu.2025.1620365

**Published:** 2025-07-07

**Authors:** Elizabeth Kibwana, Caroline Bundi, Domtila Kimani, Lydia Nyamako, Kelvias Keter, Agnes Mutiso, Rodney Ogwang, Duncan Bellamy, Katerina Rapi, Amelia Bajer, Samuel Provstgaard-Morys, Lisa Stockdale, Olivia Munoz, Mehreen S. Datoo, Alison Lawrie, Fernando Ramos-Lopez, Rachel Roberts, Mainga Hamaluba, Adrian V. S. Hill, Philip Bejon, Katie J. Ewer, Melissa Kapulu

**Affiliations:** ^1^ Centre for Geographic Medicine Research, Coast, Kenya Medical Research Institute-Wellcome Trust Research Programme, Kilifi, Kenya; ^2^ The Jenner Institute, University of Oxford, Oxford, United Kingdom; ^3^ Centre for Tropical Medicine and Global Health, Nuffield Department of Medicine, University Oxford, Oxford, United Kingdom

**Keywords:** malaria, R21/Matrix-M, controlled human malaria infection, antibodies, complement fixing antibodies, memory B cells, T follicular helper cells

## Abstract

**Introduction:**

The recently licenced R21/Matrix-M vaccine induces a protective antibody response. In this study, we examined vaccine-induced responses in semi-immune adults in a controlled human malaria infection (CHMI) Phase IIb clinical trial.

**Methods:**

Plasma and peripheral blood mononuclear cells from healthy adult volunteers living in coastal Kenya were analysed following vaccination with R21/Matrix-M (*n* = 19) and CHMI challenge with *Plasmodium falciparum* (*Pf*SPZ NF54) sporozoites (*n* = 17). Humoral immunity was evaluated by quantifying antigen specific antibody subtypes and subclasses via ELISA, alongside functional antibody properties including avidity and complement fixation elicited by vaccination and challenge. Antigen-specific memory B cells were characterised using FluoroSpot assays to detect concurrent secretion of multiple antibody isotypes and the frequency and phenotypes of circulating Tfh (cTfh) cells were assessed using multiparametric flow cytometry.

**Results:**

Vaccination increased antibody titres across IgA, IgM, and IgG isotypes and IgG1 and IgG3 subclasses but not IgG2 or IgG4 subclasses, targeting different vaccine antigens (full-length R21, NANP, and C-terminus), indicating a broad and heterogeneous response. The responses were maintained over time and, importantly, they demonstrated complement-fixing capabilities. IgG+ and IgA+ antigen-specific memory B cells were boosted but were short-lived for IgA. We observed an increase in total CXCR5+/PD1+ cTfh cells following vaccination and challenge with the predominant Th2/Th17 population.

**Discussion:**

We provide insights into the diverse immune responses induced by R21/Matrix-M vaccination and their potential contribution to protection against malaria. These findings highlight the potential of the R21/Matrix-M vaccination and protection in adults with varying levels of prior malaria exposure.

## Introduction

Malaria remains a global health challenge despite significant progress in reducing the burden of disease (1). The World Health Organization (WHO) has recommended two malaria vaccines for use in children: RTS,S/AS01 in 2022, and R21/Matrix-M in 2023 (2). These vaccines represent a turning point in the fight against malaria. However, critical gaps remain in our understanding of the mechanisms of protection and methods to improve their effectiveness in different population groups.

R21/Matrix-M, the second malaria vaccine to receive WHO endorsement, builds on the design of RTS, S and demonstrates high anti-NANP immunogenicity and high vaccine efficacy (3, 4). Both vaccines target the *P. falciparum* circumsporozoite protein (CSP), a key surface protein of sporozoites (5). CSP is composed of an N-terminal region, a highly repetitive central region containing NANP and NVDP motifs, and a C-terminal region. The central region of NANP contains key B-cell epitopes that are responsible for inducing protective antibody responses (6). R21/Matrix-M induces strong and sustained anti-NANP IgG responses in both children and adults, which is associated with vaccine efficacy. The NANP repeat region is the primary immunological readout in clinical trials as such immunogenicity studies have primarily focused on measuring total NANP-specific IgG levels (3, 4, 7). The specific antibody features (affinity, avidity, and half-life) required for robust protection are currently unknown. Moreover, the cellular immune mechanisms that underpin these humoral responses, particularly the roles of T follicular helper (Tfh) cells and memory B cells (MBCs), have not been thoroughly investigated.

A key component of vaccine-induced immunity is the interaction between B cells and Tfh cells, a specialised subset of CD4+ T cells that orchestrate antibody responses within germinal centres (GC) (8). Tfh cells support B-cell differentiation and maturation, as well as antibody isotype class switching, while generating high-affinity antibodies and both long-lived plasma cells and MBCs (8, 9). While primarily found within GC, a subset of CD4+ T cells (cTfh) that circulate in peripheral blood shows functional, phenotypic, and transcriptional similarities with their GC counterparts, including the expression of chemokine receptor CXCR5 and inhibitory receptor programmed cell death (PD-1) (10–12). These cells can act as proxies for investigating GC responses *ex vivo* and can serve as valuable biomarkers for assessing vaccine-induced immunity. Research on how R21/Matrix-M vaccination affects cTfh cell dynamics, MBC formation, and immune memory durability remains limited. Understanding these mechanisms is essential for improving R21/Matrix-M and guiding next-generation malaria vaccine development which provides lasting protection, particularly in low-transmission settings where natural boosting is infrequent. Successful global malaria control depends on an understanding of the immune processes required to broaden vaccine efficacy and refine malaria vaccine approaches.

Here, we sought to characterise both humoral and cellular immune responses to R21/Matrix-M in adult vaccinees in Kenya. Humoral responses were assessed based on antibody titres, isotypes, subclasses, avidity, and complement fixation against CSP antigens, including post-challenge boosts. Cellular immunity focused on antigen-specific MBCs and circulating cTfh cell kinetics. The booster doses were evaluated for their effects on immune responses, additionally the effects of pre-existing malaria immunity and sporozoite challenge on vaccine-induced responses were also examined. The evaluation of R21/Matrix-M vaccine effectiveness depends on a thorough characterisation of the vaccine induced immune response which helps to identify immune correlates that reveal how protection is achieved.

## Materials and methods

### Immunization groups, plasma and peripheral blood mononuclear cells

Plasma samples and peripheral blood mononuclear cells (PBMCs) were obtained from a Phase IIb sporozoite infection trial at the KEMRI Wellcome Trust Research Programme in Kilifi, Kenya (13). The plasma and PBMC samples from the trial were all prepared and frozen as previously described (14). The trial assessed the safety, immunogenicity, and protective efficacy of R21/Matrix-M and ChAd63-MVA encoding ME-TRAP using a CHMI model. A total of 56 volunteers were randomized into one of three groups - two groups of volunteers received three doses of 10μg R21 in 50 μg Matrix-M, administered one month apart, and the last group was the unvaccinated control group. The 56 volunteers were divided into 2 cohorts, the first cohort participated in CHMI, while the second cohort did not. Four weeks after the last vaccination, two groups received intradermal (ID) inoculation with 22,500 aseptic, purified, and cryopreserved PfSPZ (Sanaria ^®^ PfSPZ Challenge): R21 vaccinees (*n* =12) and unvaccinated controls (*n* = 9). The second group of R21 vaccinees (*n* = 5) received direct venous inoculation (DVI) with 3,200 PfSPZ (**Supplementary Figure 1**). Monitoring of parasitaemia via qPCR was conducted as described in (15), at three time points: pre-vaccination, one day prior to challenge (to confirm absence of infection) and during post-challenge monitoring. Malaria management followed standardized protocols upon qPCR positivity. Full details of qPCR methodology, inclusion/exclusion criteria, and clinical monitoring procedures are comprehensively described in the associated clinical trial manuscript (13).

All approvals required for the trials were obtained as previously described (13), and the trial was registered on ClinicalTrials.gov identifier NCT03947190. Written informed consent was obtained from all volunteers prior to participation. The study adhered to the principles outlined in the Declaration of Helsinki and complied with Good Clinical Practice guidelines. All participants consented to the use of their samples for exploratory immunological analysis.

### Antigens used in the study

All CSP-based assay antigens were kindly donated by the Jenner Institute at the University of Oxford. Multi- and single-plex CSP ELISAs as well as antigen-specific FluoroSpot assays were performed using a synthetically produced peptide consisting of six NANP repeats and a cysteine residue (NANP)6C (Think Peptides, ProImmune, Oxford, UK), synthetically produced full-length R21 (Serum Institute of India) and C-terminus (thinkpeptides, ProImmune, Oxford, UK). Sequence information for these antigens is provided in **Supplementary Table 1**. Recombinant full-length MSP1 was expressed in mammalian cells and generously provided by Dr James Tuju (KEMRI-Wellcome Trust, Kilifi) (16), while schizont extract was prepared in-house by cultivating parasite cultures and isolating schizont extract using MACS beads as described by (17, 18).

### CSP ELISAs

The plasma samples were tested for anti-CSP responses using a standardised ELISA protocol adapted from a previous study (7). Briefly, 96-well NUNC Immuno plates (Fisher) were coated with 50 μl of 0.2 ug/ml of NANP6C or 1.5 μg/ml of full-length R21 or C-terminus in carbonate bicarbonate coating buffer (Sigma) and incubated overnight at 4°C. The plates were washed with PBST (Sigma), blocked with 100 μl of casein (Thermo Scientific) for one hour at RT, and then washed again. Plasma samples (50 µL; 1:500 for day 0, 1:1000 post-vaccination) diluted in casein were added in triplicates and incubated for 2 h at RT. After washing, the plates were incubated with 50 μl of goat anti-human IgG (γ-chain) conjugated to alkaline phosphatase ((AP) (Sigma)) diluted at 1:1000 for 1 h at RT. Following a final wash, plates were developed with 50 μl of p-nitrophenyl phosphate (pNPP (Sigma)) at 1 mg/mL in diethanolamine buffer ((DEA), (Pierce)) and optical density (OD) was read at 405 nm using a Bio-Tek ELx800 Microplate Reader with Gen5 software. Standard curves were generated from a pool of hyperimmune sera vaccinated with R21/Matrix-M from a previous study (3) and included on each plate along with a positive pool of hyperimmune sera (PHIS) and four individual UK malaria-naïve controls (negative controls); all tests were performed in triplicate to ensure accuracy and consistency. The standard curves allowed for interpolation of antibody ODs in arbitrary units. The ELISA detection limit was OD 0.2. Values below this threshold were assigned an AU of 11 for R21, 14 for NANP, and 41 for the C-terminus. The seropositivity cut-off was defined as three times the mean OD of naïve sera plus the standard deviation; samples exceeding this value were considered seropositive.

### Antibody isotype ELISA

IgA and IgM CSP isotype ELISAs were conducted using the same procedure as the total CSP IgG ELISAs, including plate coating, incubation periods, washing steps, and standard curves and control samples, except that all serum samples were diluted 1:500 in casein. Following a 2-hr incubation at RT, the plates were washed, and anti-human IgA alkaline phosphate (Sigma, 1:1000) or anti-human IgM alkaline phosphate (Sigma, 1:1000), both diluted in casein, were added. IgG subclass ELISA was conducted in a similar manner. The IgG subclasses were detected using mouse anti-human IgG1/IgG2/IgG3/IgG4 antibodies conjugated to AP (Invitrogen) at 1/1000 dilution for 1 h at RT, the plates were developed with pNPP (Sigma)/DEA (Pierce)) and OD was read at 405 nm.

### Schizont and MSP-1 ELISAs

Anti-*Plasmodium* ELISAs were performed exactly as the CSP ELISA using purified synthetic MSP-1 at a coating concentration of 2 µg/ml and *P. falciparum* NF54 schizont lysate at 1:5000 dilution. All subsequent steps were conducted described in the CSP ELISAs above. Standard curves were generated from a pool of hyperimmune adult sera challenged with sporozoites in the CHMI-SIKA clinical trial (15, 19).

### CSP IgG antibody avidity ELISA

The avidity of the vaccine-induced antibodies was assessed using the sodium thiocyanate (NaSCN)-displacement ELISA method outlined by Biswas et al., 2014 (20). Briefly, plasma samples were normalised individually in casein to achieve an OD405 of 1.0, ensuring equivalent antigen-specific IgG titres. The plates were coated overnight with antigen and blocked the next day with casein. Samples at OD405 of 1.0 (50 μl/well) were incubated for 2 h at room temperature (RT). An NaSCN gradient (0M to 7M) was prepared from an 8 M stock solution in PBS, added to duplicate wells, and incubated at RT for 15 min. After incubation and washing, a secondary antibody was added, and the plates were developed according to the total IgG ELISA protocol. The avidity index was calculated based on the NaSCN concentration required to decrease the OD405 by 50% compared to wells without NaSCN.

### CSP complement ELISA

ELISA plates were coated with 50μl of either purified NANP6C (0.2 μg/ml) or the C-terminus (1.5 μg/ml) and incubated overnight at 4°C. The plates were washed four times with PBS and blocked with 200 μl of casein for two hours at 37°C. Plasma samples (50μl, diluted 1:100 in casein) were added to the plates and incubated for 2 h at 37°C. Plates were washed and 40μl of recombinant human complement C1q (Calbiochem) diluted to 10 μg/ml in casein was added and incubated for 30 min at 37°C. Following another wash, 50 μl anti-C1q antibody conjugated to horseradish peroxidase (HRP) at 1:100 in casein (Abcam) was added and incubated for 1 h at 37°C, followed by a final wash. Development was performed using 50 μl of the OPD substrate for 30 min at RT and the reaction was stopped with 2 M H_2_SO_4_. ODs were measured at 492 nm using a Bio-Tek Gen5 v3.00 ELISA reader.

### Meso Scale Discovery immunoassay

This assay was performed as previously described (21). Assay plates were pre-coated by MSD, USA, with four vaccine antigens (full-length R21, NANP, Cterm, and HBsAg) on independent spots, and stored at 4°C until required. Plates were brought to RT, blocked with casein for 30 min, and washed three times with PBST before blotting. A reference standard control was prepared for the standard curve and positive controls (high, medium, and low) were prepared. Fifty microlitres of the reference standard (pooled adult hyperimmune sera), positive controls, and diluted samples were added to the plates and incubated for 2 h at RT with shaking. After washing, 50 μl/well of the SULFO-TAG detection antibody (100 μg/ml) was added and incubated for 1 h at RT with shaking. Following the final wash, 150 μl/well of MSD GOLD buffer was added to activate the electrochemiluminescence reaction. Plates were read immediately using the MSD multiplex instrument and analysed using the MSD Methodical Mind Software.

### Antigen specific memory B cell FluoroSpot assay

#### Preparation and stimulation of PBMCs

The assay was conducted as described by Jahnmatz et al. (22). Frozen PBMCs were thawed, rested, counted, and adjusted to 250,000 cells/200 μl. The cells were stimulated with 1 μg/mL resiquimod (R848) and 10 ng/mL recombinant human IL-2 (both from Mabtech) in R20 culture media (RPMI with 20% FCS, 100 U/mL penicillin, 10 mM HEPES, 100 μg/mL streptomycin, and 2 mM L-glutamine) and 50 U/mL benzonase endonuclease (Novagen) for 5 days at 37°C and 5% CO2, allowing MBCs to differentiate into antibody-secreting cells (ASC). After incubation, the cells were harvested, washed by centrifugation (1800 rpm for 5 min), and resuspended to a final concentration of 250,000 cells/100 μl per well.

#### FluoroSpot assay

Low-fluorescent PVDF plates (Mabtech) were activated with 15 μl/well of 30% ethanol for 1 min, washed five times with distilled water, and coated overnight at 4°C with 100 μl of (i) full-length R21, (ii) C-terminus, (iii) capture monoclonal antibodies (IgG, IgA, and IgM (positive control)), and (iv) PBS (negative control) in duplicate wells. The next day, the plates were washed with PBS, blocked with 100 μl of R10 (RPMI containing 10% FCS, 100 U/mL penicillin, 10 mM HEPES, 100 μg/mL streptomycin, and 2 mM L-glutamine) at 37°C for 1 h, and 250,000 stimulated cells/100 μl/well were added to antigen and negative control wells. The positive control wells received 50,000 cells/100 μl. The plates were incubated at 37°C and 5% CO2 for 20 h. After washing, 100 μl of conjugated detection mAb (anti-human IgG-550, anti-human IgA-490, and anti-human IgM-640) was added to all wells, incubated for 2 h, washed five times, and the fluorescent enhancer was added for 5–10 minutes. Plates were air-dried overnight in the dark, and spots were analysed using an IRIS FluoroSpot Reader with the Apex software (Mabtech). Positive wells were identified based on the presence of antigen-specific ASC. MBC responses were quantified as the percentage of antigen-specific spots per total IgG, IgA, or IgM level. Assays were not performed if fewer than 3 million cells were obtained. Due to the unavailability of key reagents, we were unable to assess NANP specific memory B cell responses.

### Flow cytometry

Surface phenotyping of cTfh cells was performed on thawed cryopreserved PBMCs in 96-well V-bottom plates as previously described (23). Briefly, 1.5 × 10^6^ PBMCs in 200 μl were washed twice with FACS buffer (PBS containing 0.1% BSA and 0.01% sodium azide). Cells were stained with LIVE/DEAD Aqua dye (Life Technologies) for 20 min at RT in the dark, washed, and incubated with 50 μl of Tfh surface staining antibody cocktail (**Supplementary Table 2**) for 30 min at 37°C. After washing, the cells were fixed with 1% paraformaldehyde (PFA) and acquired using a BD LSRFortessa cell analyser (BD Biosciences). Compensation for AQUA was performed using single-stained One-Comp beads and ARC beads. Flow cytometry data were analysed using FlowJo v10.10.0 (Tree Star). The antibody catalogue numbers and clones are summarised in **Supplementary Table 2**.

### Statistical analysis

All datasets were non-normally distributed and were analysed using non-parametric methods in GraphPad Prism 10.1.2. Antibody endpoint titres were interpolated from standard curves and reported as geometric mean titres (± 95% confidence interval (CI)) in ELISA antibody units (AU). IgG subclass data are presented as raw optical densities (ODs) and as medians with interquartile ranges. Mann Whitney test was used when comparing the median of two distinct groups. Owing to participant dropout (17.2%–25.1%, depending on the time point and assay), insufficient PBMCs, and variable sample sizes, the Kruskal–Wallis test replaced the Friedman test for unpaired datasets. Subset analysis using the Friedman test yielded consistent results, confirming the robustness of the results. Statistical significance was set at p < 0.05. For the avidity index analysis, only samples with positive antibody titres (as measured by ELISA) were used. Correlations were assessed using Spearman’s rank, and the significance level was set at p < 0.05.

## Results

### Study design and baseline demographic characteristics

Between June and August 2022, 56 healthy adults aged 18–45 years were enrolled and randomised into three groups (**Supplementary Figure 1**). The mean age of the participants was 28 years (standard deviation [SD] = 6), and the majority were male (71%). The detailed study design and safety assessment have been described by Kapulu et al. (13). Demographic and baseline characteristics are shown in **Supplementary Table 3**.

### Evidence of prior malaria exposure

To measure prior malaria exposure, we measured the initial antibody responses at baseline to MSP-1 (an immunogenic blood-stage antigen) and NF54 schizont extracts in 47 participants (9 individuals were inadvertently excluded from this analysis because of insufficient reagent availability). Most participants exhibited pre-existing immunity, with 59.6% (28/47) testing positive for schizont-specific antibodies and 72.3% (34/47) showing MSP-1 responses (**Supplementary Figures 3A, B**). MSP-1 and schizont-specific antibody responses were significantly correlated (*r* = 0.58, *p* = 0.001, n = 47).

### R21/Matrix-M vaccination induces robust and durable IgG antibody responses to CSP vaccine components with no additional boost from CHMI

**Figure 1:**
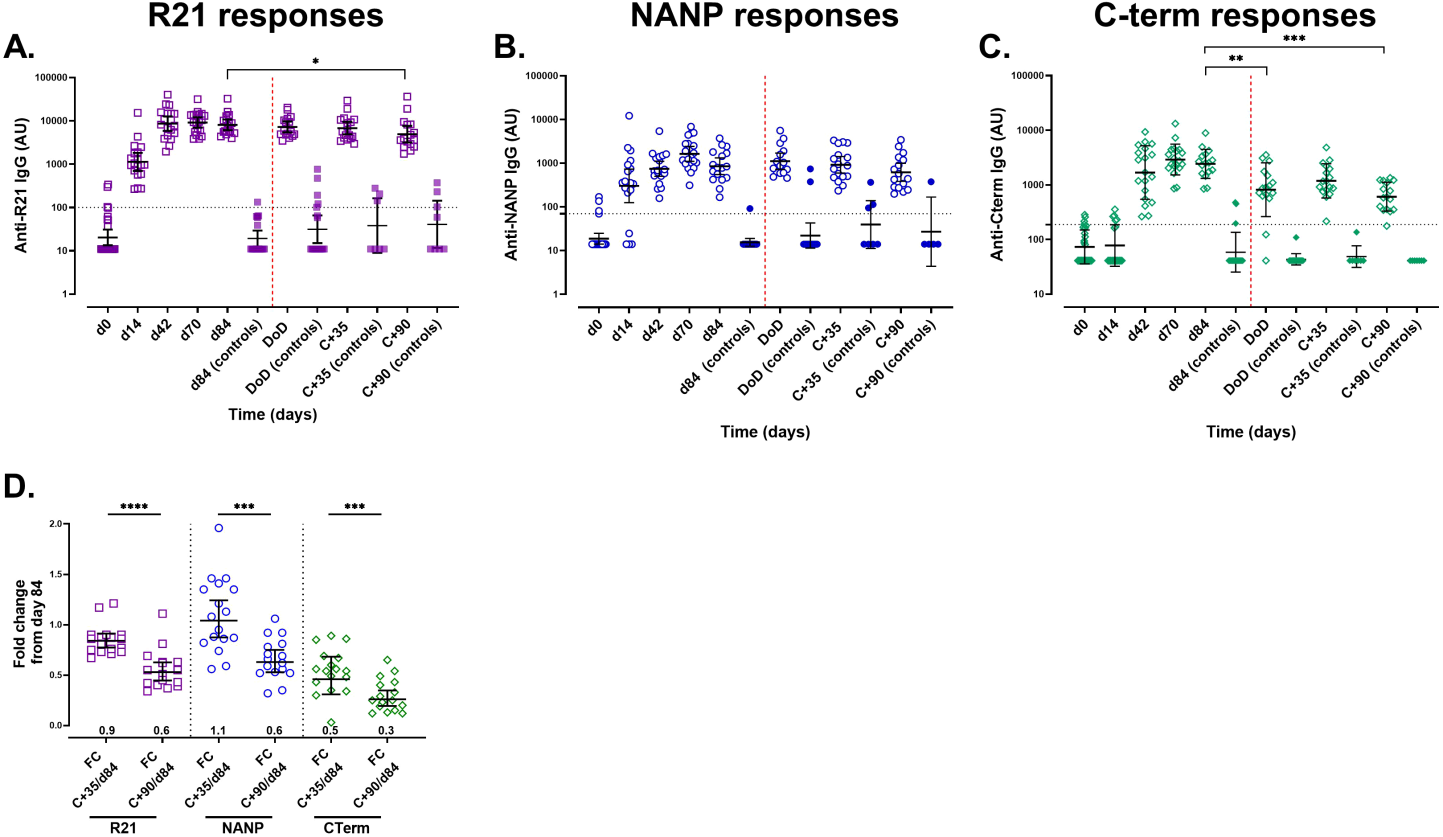
Anti-IgG responses measured by a single-plex ELISA assay following vaccination with R21/Matrix-M and sporozoite challenge. The kinetics of the total IgG responses were analysed over time from pre-vaccination (day 0) to day 84 (i.e. one month after the 3^rd^ booster dose) using standardised single-plex ELISAs. Following CHMI, antibody responses were monitored on the day of diagnosis (DoD), at C+35, (35 days post-challenge), and C+90, (90 days post-challenge) post-CHMI. **(A)** Anti-full-length R21 responses, **(B)** anti-NANP responses, and **(C)** anti-C-terminus. **(D)** Antibody durability measured by the fold change between days 84 and C+35 and C+90. Geometric mean antibody titres (GMT ±95% CI) are presented as log10 AU. The positivity threshold, indicated by a horizontal dashed line, represents the mean + 3x standard deviations of U.K. malaria-naïve (negative controls). Samples that exceeded this threshold were classified as positive samples. The vaccination time points are marked by vertical black dashed lines **(A-C)**. Sporozoite challenge is marked by a red vertical dashed line. The ELISA detection limit was OD 0.2; values below this limit were assigned as AU values of 11 for R21, 14 for NANP, and 41 for the C-terminus. Open symbols represent vaccinees (n = 19 or 17 (from day 84)) and filled symbols represent unvaccinated controls (n = 9) **(A-C)**. *P < 0.05, **P < 0.01, ***P < 0.001, ****P < 0.0001.

Vaccination induced strong total IgG antibody responses against all three CSP vaccine components (full-length R21, NANP, and C-terminus) as measured by single-plex ELISA (**Figure 1**) and corroborated by multiplexed MSD assays (**Supplementary Figure 4**). IgG antibody titres increased with each dose, peaking on day 70, two weeks after the final dose. All participants were seronegative for CSP-specific responses at baseline. Responses to HBsAg remained low, with only a modest increase observed after vaccination (**Supplementary Figure 4**). Correlation analysis revealed that previous malaria exposure had little impact on vaccine-induced antibody responses, as similar IgG levels were observed regardless of the baseline malaria serostatus. This suggests that R21/Matrix-M can effectively stimulate immune responses even in individuals with pre-existing immunity to malaria.

**Figure 2:**
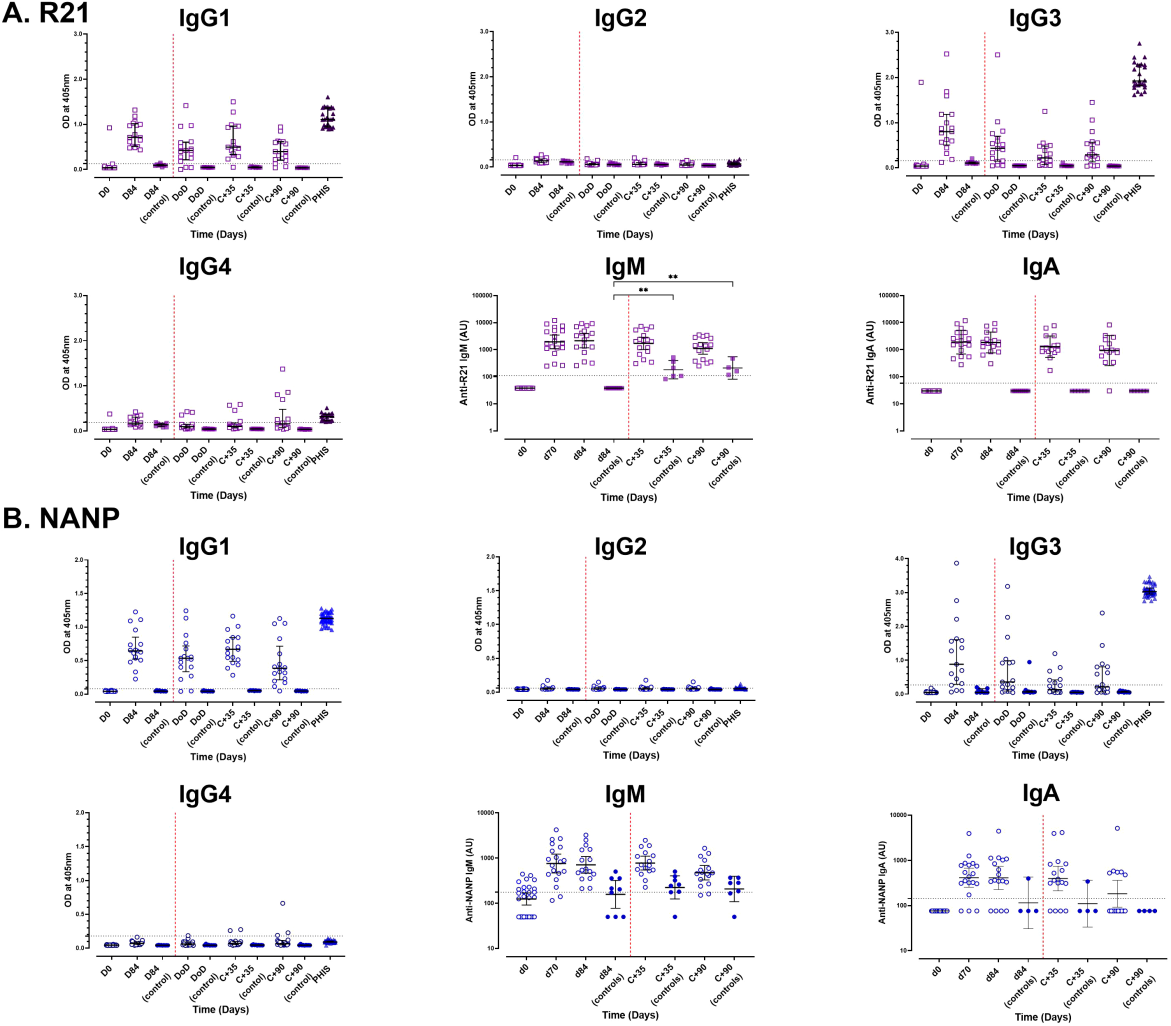
Analysis of antibody isotype profiles to full-length R21 and NANP following vaccination and sporozoite challenge. Vaccine induced antibody isotype profiles were analysed using single-plex ELISA at baseline, day 84 (one-month post the third dose), and 35 (C+35) and 90 (C+90) days post-CHMI. **(A)** Full-length R21 and **(B)** NANP responses. Antibody subclass (IgG1-4) levels are presented as raw ODs at 405 nm, shown as individual responses along with the median [± IQR] values, whereas IgM and IgA subclasses were converted to arbitrary units (AU) and are shown as GMT (± 95% CI) in log10 AU. The seropositivity threshold, indicated by a horizontal dashed line, represents the mean + 3x standard deviations of U.K. malaria-naïve negative controls, with values above this threshold classified as positive. Sporozoite challenge is marked by a red vertical dashed line. PHIS, pooled adult hyperimmune sera (positive controls). **P < 0.01.

Following either ID (*n* =12) or DVI *(n* =5) sporozoite challenge (i.e. from day 84 to 90 days post-challenge), all R21/Matrix-M vaccinees (*n* = 17) showed a gradual decline in total IgG titres against all three CSP vaccine components (**Figures 2A-C**), suggesting that parasite exposure did not significantly boost vaccine-induced responses. Fold-change analysis validated the reduction in antibody titres from day 84, demonstrating waning of immune responses and no boosting from the challenge (**Figure 2D**). The antibody titres in the unvaccinated controls did not change (*n* = 9).

### R21/Matrix-M vaccination elicits robust antibody responses across isotypes and IgG1/IgG3 subclasses

Vaccination significantly increased antibody isotype responses to full-length R21 and NANP, dominated by the IgG1 and IgG3 subclasses. Anti-full-length R21 IgG1 (median OD: 0.04 [IQR: 0.036–0.041, *n* = 19] and IgG3 (0.038 [IQR: 0.036–0.041, *n* = 19]) at baseline rose to 0.710 [IQR: 0.51–1.01, *n* = 17] and 0.800 [IQR: 0.504–1.185, *n* = 17], respectively, by day 84. Similarly, NANP-specific IgG1 increased from median OD: 0.043 [IQR: 0.042–0.047, *n* = 19] to 0.645 [IQR: 0.522–0.850, *n* = 17], and IgG3 from 0.048 [IQR: 0.046–0.058, *n* = 19] to 0.881 [IQR: 0.288–1.608, *n* = 17] (**Figure 2**).

IgM and IgA antibody responses also increased following vaccination, with R21 IgM titres peaking on day 84 (GMT 2155; 95% CI: 1174–3954, *n* =17) and IgA titres peaking on day 70 (GMT 1908; 95% CI: 1174–3103, *n* =19). NANP IgM and IgA titres peaked on day 70, GMT 757.9 (95% CI: 469.6 – 1223, *n* = 19) and GMT 413.8 (95% CI: 254.3 – 673.3, n = 19), respectively (**Figure 2**).

Similar to the total IgG responses, we observed no significant boosting of vaccine-induced IgG subclasses or IgM and IgA isotypes in vaccinated individuals following sporozoite challenge. However, the unvaccinated controls showed increased IgM levels to both full-length R21 and NANP, this was significant to full-length R21 (day 84 vs C+35 and vs C+90, *p* < 0.002), likely indicating a primary immune response to sporozoite challenge.

### Vaccine-induced antibody responses enable complement fixation and display diverse avidity

**Figure 3:**
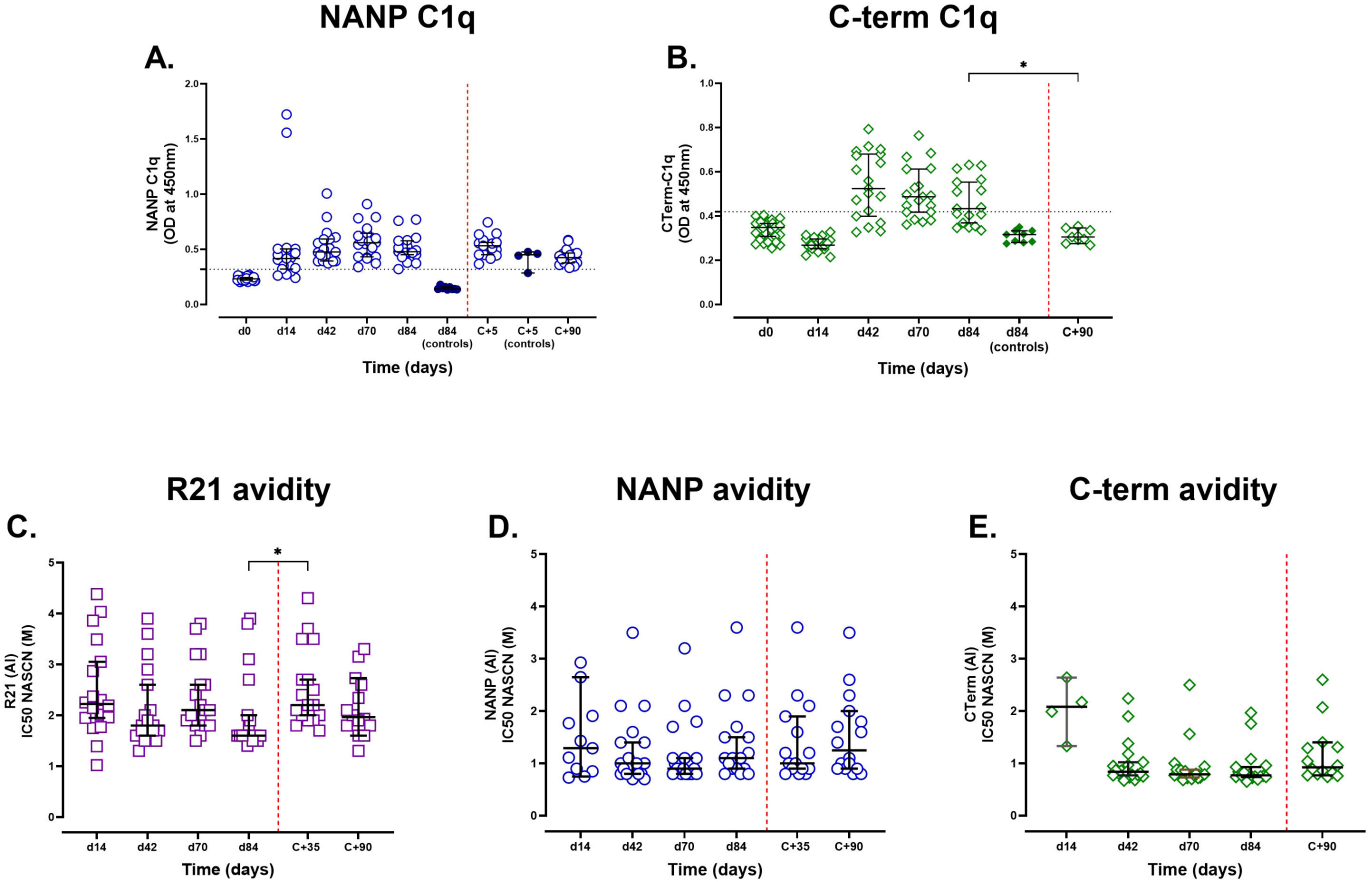
Assessment of complement fixation activity and IgG avidity post-R21/Matrix-M vaccination and sporozoite challenge. Semi-immune adults vaccinated with R21/Matrix-M were tested for C1q fixation by NANP and C-terminal antigens (**A** and **B** respectively). Individual and median [IQR] OD_450nm_ responses are shown. The positivity cutoff was the mean plus three standard deviations of day 0 (horizontal dashed line). The avidity index was determined as the molar concentration of NaSCN required to reduce OD405 to 50% of the value observed in the absence of NaSCN. **(C)** Avidity to full-length R21, **(D)** avidity to NANP, and **(E)** avidity to the C-terminus. Samples with negative antigen-specific total IgG responses by ELISA or insufficient antibody levels for avidity testing at a given time point were excluded from the analysis. Individual avidity responses are displayed alongside the group median and ±95% CI to illustrate variability and overall trends in avidity over time. The Kruskal-Wallis non-parametric test with Dunn’s multiple comparisons was used to assess differences between time points. The challenge day is represented by the red dashed vertical line. *P < 0.05.

To assess functional antibody activity, we measured complement fixation (C1q-binding) (**Figures 3A, B**). All vaccinees (100%, 19/19) developed C1q-binding NANP antibodies by day 42 after the first dose, peaking on day 70 before declining. In contrast, C-terminus-specific C1q-binding antibodies emerged post-dose 2, but were transient (68.4% positive at day 42; 52.9% at day 84). Unlike NANP, not all vaccinees developed C1q-binding C-terminus responses (**Figure 3B**).

Post-challenge, NANP antibodies in vaccinees gradually declined but remained above seropositivity at C+90, whereas unvaccinated controls showed an increase in NANP responses (*n* = 4), likely from natural priming. C-terminus antibodies returned to baseline by C+90.

On day 84, C1q-binding strongly correlated with total IgG for both NANP (*r* = 0.79, *p* = 0.0003, *n* = 17) and C-terminus (*r* = 0.77, *p* = 0.0004, *n* = 17) and with IgG1/IgG3 subclasses (NANP: IgG1, *r* = 0.77, IgG3 *r* = 0.84; *p* < 0.001, *n* = 17), consistent with their role in complement activation.

To assess whether vaccination influenced antibody avidity, we measured IgG binding strength using NaSCN-displacement ELISA on days 14, 42, 70, and 84 (**Figures 3C-E**). Avidity varied widely, particularly towards full-length R21 and NANP, but remained largely unchanged following additional vaccinations, suggesting that although antibody levels increased, their binding strength did not improve significantly after each vaccination. Avidity to the C-terminal region was more consistent across individuals and time points, with minimal changes observed throughout the vaccination and challenge period, suggesting limited affinity maturation. Notably, avidity to full-length R21 increased significantly post-challenge, suggesting that parasite exposure may enhance antibody functional quality (**Figure 3C**).

We also investigated whether there was a relationship between vaccine-induced antibodies and avidity on day 84. While there was little association between avidity and vaccine-induced anti-full-length R21 and anti-NANP antibody levels, anti-C-terminus IgG antibodies exhibited a significant negative association with C-terminus avidity (*r* = -0.511, *p* = 0.038, *n* = 17), suggesting that the less immunodominant properties of the C-terminus region might favour the production of a larger proportion of low-affinity antibodies.

### Vaccination induces antigen-specific memory B cells and expands circulating Tfh cells

**Figure 4:**
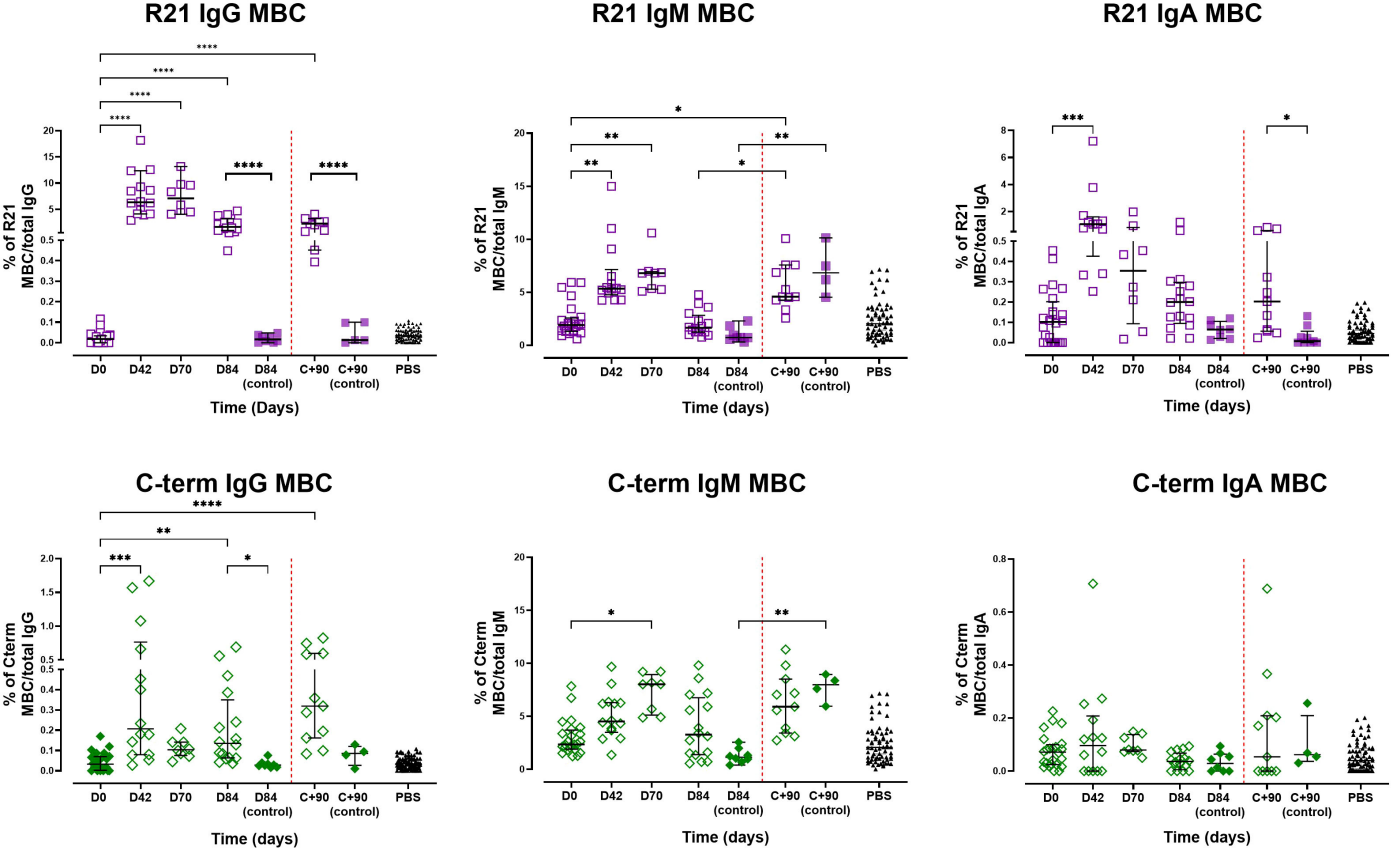
Memory B cell responses following vaccination and sporozoite challenge. The magnitude of MBC responses to full-length R21 and the C-terminus was measured using B cell FluoroSpot in pre-exposed adults vaccinated with R21/Matrix-M. Responses were measured after vaccination and post-CHMI. Top panel: Proportion of full-length R21-specific MBCs relative to total IgG-, IgM-, and IgA-producing cells. Bottom panel: Proportion of C-terminus-specific MBCs relative to total IgG-, IgM-, and IgA-producing cells. The magnitude of MBC responses was expressed as the proportion of antigen-specific spots per total IgG/A/M, expressed as a percentage (% of antigen-specific MBC/total IgG, IgA, or IgM). Individual and group medians [± IQR]) are shown. The Kruskal-Wallis non-parametric test with Dunn’s multiple comparisons was used to assess differences between time points. Challenge day is represented by a red dashed vertical line. *P < 0.05, **P < 0.01, ***P < 0.001, ****P < 0.0001.

Full-length R21-specific IgG MBC frequencies increased post-vaccination, peaking on day 70 (median 7.09%, *n* = 8) before declining on day 84 (median 1.63%, *n* = 16). Similarly, IgM and IgA MBCs increased, peaking on days 70 and 42, respectively. IgG, IgA, and IgM MBC in unvaccinated controls remained low and unchanged following vaccination (**Figure 4** – top panel). C-terminus-specific IgG and IgM MBCs increased from baseline to peak on days 42 and 70 for IgG and IgM, respectively. The C-terminal MBC IgA response remained largely unchanged following vaccination (**Figure 4** – bottom panel).

At C+90, the frequencies of full-length R21- or C-terminus-specific IgG MBCs in vaccinated individuals showed a non-significant increase compared with pre-challenge levels (day 84), suggesting a modest challenge-induced boost in antigen-specific responses. The unvaccinated controls maintained low antigen-specific IgG MBC frequencies post-challenge, indicating that sporozoite exposure alone was insufficient to drive the expansion of these antigen-specific populations without prior vaccination. Conversely, IgM MBCs for both antigens increased significantly post-challenge in all participants (vaccinated, *p* = 0.017; unvaccinated, *p* = 0.002), potentially reflecting primary responses in controls and naive B-cell recruitment or reactivation of existing IgM MBCs in vaccinees. Full-length R21-specific IgA MBCs showed no change from day 84 to C+90 but remained significantly higher compared to unvaccinated controls, while C-terminus-specific IgA MBCs were similarly unchanged, with no significant difference between vaccinated and control groups at C+90. We noted non-specific binding to the antigen-free wells (PBS coated wells), particularly IgM MBCs, likely due to the polyreactive nature of IgM antibodies, which are known to exhibit low-affinity interactions with various targets and bind non-specifically to the plate wells.

Spearman analysis revealed no significant correlation between full-length R21 or C-terminus-specific IgG MBC frequencies and total IgG antibody levels on day 84 or C+90, despite similar kinetics. IgA/IgM MBCs also showed minimal association with IgA/IgM antibody titres. However, full-length R21 specific IgM antibodies at C+90 correlated positively with full-length R21 specific IgM MBC frequencies on day 84 (*r* = 0.557, *p* = 0.03, *n* =15).

**Figure 5:**
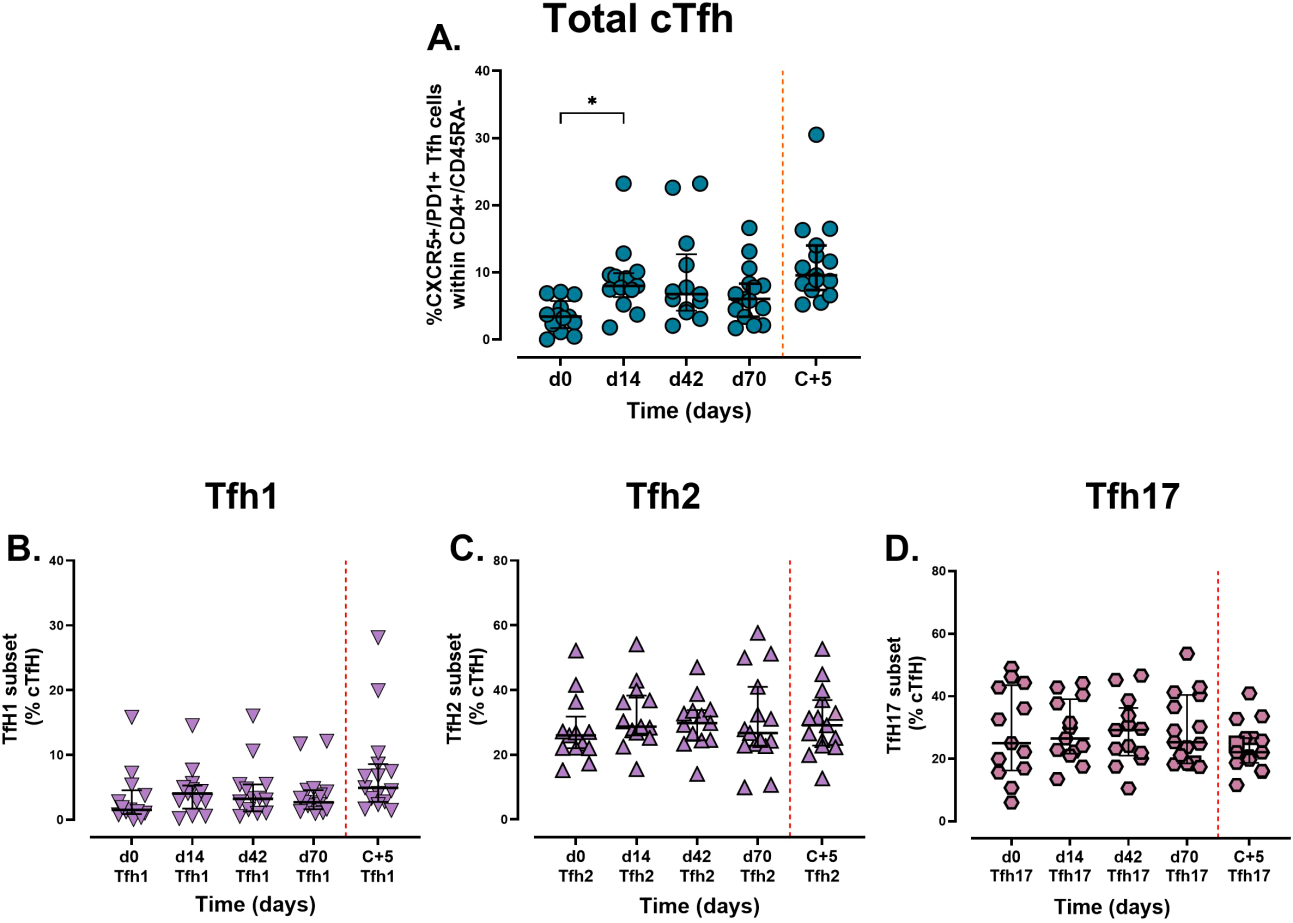
Analysis of circulating Tfh cells and subsets following vaccination and sporozoite challenge. Cryopreserved PBMCs from days 0, 14, 42, and 70 were stained *ex vivo* and analysed using flow cytometry. Circulating Tfh cells are defined by the expression of **(A)** CXCR5+/PD1+. Tfh were further analysed into subsets **(B)** Th1 (CXCR3+/CCR6-), **(C)** Th2 (CXCR3-/CCR6-), and **(D)** Th17 (CXCR3-/CCR6+). Data are presented as individual points and grouped median [ ± IQR]. The Kruskal-Wallis non-parametric test with Dunn’s multiple comparisons was used to assess differences between time points. *P < 0.05.

The frequency of non-vaccine-specific cTfh (CXCR5+/PD1+, see **Supplementary Figure 2** for gating) increased significantly from 3.4% [IQR: 1.7 – 5.7, *n* = 13] on day 0, to 8.0% [IQR: 6.3 – 9.9 *n* = 13) on day 14, *p* = 0.005) (**Figure 5A**), suggesting that vaccination induced Tfh activation which are critical for supporting antibody production by B cells. No further activation was observed with additional doses, although challenge did boost the frequency of total cTfh from 6.0% [IQR: 3.4 – 8.3, *n* = 15] at day 70 to 9.6% [IQR: 7.4 – 14, *n* = 15] at 5 days post challenge. No significant differences were observed in the frequency of each subset (Th1/Th2 and Th17 - **Figures 5B–D**). Th2 and Th17 subsets remained the dominant cTfh subsets, whereas Th1 frequencies persisted at low levels despite minor increases post-vaccination and challenge.

Spearman analysis revealed a moderate positive correlation between day 14 cTfh frequencies and day 84 full-length R21 IgG MBCs (*r* = 0.577, *p* = 0.043, *n* =13), suggesting that cTfh is involved in B cell memory activation/maintenance. No associations were observed with C-terminus MBCs or total antibody titres. However, these findings are limited by the small cohort size.

## Discussion

We show that R21/Matrix-M elicits high IgG titres against the immunodominant NANP repeats and full-length R21 antigen after a single dose, while anti-C-terminus antibodies required two doses for significant induction. The C-terminal region includes an α-thrombospondin repeat (αTSR) domain, which is vital for hepatocyte invasion and contains mainly T-cell epitopes (24, 25). This likely explains the need for two doses for robust humoral responses. Functional anti-C-terminus antibodies may prevent invasion, suggesting R21/Matrix-M’s capacity to target multiple CSP regions for layered protection (24, 26). IgG subclass analysis revealed dominant IgG1 and IgG3 responses, consistent with RTS,S vaccine studies (27–29). The cytophilic IgG1 and IgG3 subclasses enhance complement activation (30), which is crucial for pathogen clearance. IgM and IgA CSP responses were also robust. Anti-parasitic IgM can activate the complement system and inhibit *Plasmodium* growth, correlating with malaria protection (31, 32). While exposure to *P. falciparum* sporozoites through infection or immunisation elicits a functional IgA response that can inhibit liver cell invasion *in vitro* (33). Our findings suggest that R21/Matrix-M induces a broad and functional antibody repertoire that may contribute to its protective efficacy.

We examined the functional capabilities of vaccine-induced antibodies, with a focus on complement fixation and avidity. Complement activation is key to pathogen clearance and has been linked to malaria protection (32, 34, 35). R21/Matrix-M elicited anti-NANP antibodies capable of fixing complement protein C1q. Lower and more transient C1q-fixing responses were elicited by C-terminus-specific antibodies, following a pattern similar to that of total IgG kinetics but declining more rapidly. A strong correlation was observed between total NANP IgG, IgG1, and IgG3 levels and C1q-fixing antibodies on day 84. Similarly, C-terminus-specific IgG titres were strongly correlated with anti-C-terminus C1q-fixing antibodies. High-avidity antibodies are generally considered to be key indicators of effective immunity, display better function, and serve as potential surrogate markers for protection (36). While the correlation between antibody avidity and malaria protection are unclear, some RTS, S vaccine studies found no association between avidity and vaccine efficacy (37, 38), whereas others reported high anti-NANP and C-terminal avidity with increased protection (39). In this study, we observed a wide range of avidity for anti-full-length R21 and anti-NANP antibodies, antibody avidity to the C-terminus region was notably lower and less variable. Minimal changes in avidity were observed following vaccination across all CSP antigens. Notably, additional vaccine doses did not enhance avidity, and one can speculate that the immune response may have reached a plateau or formed a stable response regarding antibody avidity after the first dose or from natural infection.

Prior malaria exposure, as evidenced by antibody responses to blood-stage antigens, did not significantly affect R21/Matrix-M induced humoral response. This study was conducted in a low-transmission setting, which may limit its applicability in high-transmission regions. However, an RTS, S study in a high-transmission setting showed a similar pattern, with vaccination eliciting strong anti-CS responses regardless of prior exposure (40).

Antigen-specific IgG MBCs (to both full-length R21 and C-terminus) increased after two doses but plateaued after dose 3, consistent with findings from RTS,S studies (41). This aligns with evidence that antibody feedback mechanisms suppress immunodominant epitope responses (e.g. NANP) while allowing subdominant B-cell expansion (42). Strategies such as delaying booster doses until antibody levels decline could help reduce the suppression effects and enhance B-cell activation, potentially leading to improved vaccine outcomes (33, 43, 44). IgM MBC data were confounded by assay non-specificity, highlighting the need for further optimisation of the assay or the use of improved methods for studying this population. No association was found between IgG MBCs and total IgG titres. Since MBCs take 4–7 days to produce detectable antibodies following re-exposure, they may not necessarily align with antibody levels at the same time, and measuring plasma cells may be a more suitable parameter when examining the relationship between these two factors.

R21/Matrix-M vaccination induced a significant increase in cTfh frequency by 14 days after the first dose, indicating adaptive immune activation. No further increase in cTfh number was observed with additional vaccine doses. Th2 and Th17 subsets dominate, a profile that is advantageous for humoral immunity, as these CXCR3− cTfh subsets are linked to naïve B-cell stimulation and high-affinity antibody production (12, 45, 46). We observed no significant changes in the Th1/Th2/Th17 cell subsets after vaccination. The lack of increase in Tfh subsets may stem from two factors: (i) the small sample size (*n* = 15), which likely limits the statistical power to detect subtle changes in low-frequency Tfh subsets, and (ii) the sampling timing, Tfh subsets show transient activation patterns. Chan et al. observed distinct Th2-Tfh activation during peak *P. falciparum* infection (day 8) and delayed Th1-Tfh response post-treatment (day 14/15) in malaria-naïve adults, highlighting how improper sample timing could obscure dynamic or short lived Tfh subset responses (47). While Tfh frequencies, particularly Tfh2 and Tfh17 subsets are linked to robust antibody responses (47–50), here the total Tfh/Tfh1/Tfh2 and Tfh17 frequencies at day 14 did not correlate with day 84 IgG responses to full-length R21, C-terminus, or NANP, suggesting that additional factors influence antibody production. However, the association between cTfh on day 14 and full-length R21-specific MBCs on day 84 suggests that cTfh cells may contribute to MBC generation or maintenance, supporting long-term humoral immunity. The small sample size (n = 13) limits the conclusions that can be drawn.

Although the NANP repeat region typically acts as a T-independent (TI) antigen, often associated with weak immune responses and poor memory, our study suggests that R21/Matrix-M may help address this by promoting a T-dependent-like response. The Matrix-M adjuvant effectively recruits and activates dendritic cells and Tfh cells (51), evidenced here by the significant expansion of cTfh cells 14 days following vaccination. Additionally, we also observed durable, complement-fixing IgG responses (dominated by IgG1/IgG3 subclasses) against the NANP repeat, along with antigen-specific memory B cells targeting the full-length R21 and C-terminus region of CSP. However, reagent limitations prevented us from confirming memory B cells specifically reactive to the NANP repeat region. Nevertheless, taken together, these features (Tfh expansion, durable functional IgG, and CSP-specific MBCs) are consistent with a T-dependent (TD) mechanism. This suggests the vaccine formulation and adjuvant likely promotes a substantial TD response to the CSP antigen, potentially enabling immune responses consistent with long-term protective immunity against major epitopes like the NANP repeat.

The sporozoite challenge did not boost anti-CSP antibody levels (IgG/IgA/IgM and IgG subclasses) or C1q responses in the vaccinees, with antibody titres declining gradually from day 84 to C+90. This lack of booster effect could be attributed to: (i) R21/Matrix-M immunity had reached a peak prior to the challenge, leaving limited room for further antibody amplification; (ii) insufficient antigen stimulation due to the short time sporozoites spend in circulation before reaching the liver (52, 53) this may not trigger a robust recall response; or (iii) the circulating sporozoites are rapidly cleared before inducing further immune activation. In contrast, the unvaccinated controls exhibited modest increases in IgG/IgM titres and increases in NANP complement-fixing antibodies post-challenge. Notably, IgM responses against full-length R21 were significantly elevated in controls compared to day 84. The lack of detectable anti-CSP responses at baseline in controls indicates their post-challenge antibody boosts likely arose from natural priming to CSP antigens during the challenge infection.

We observed a significant increase only in full-length R21 avidity following challenge, suggesting that the antibodies may have undergone further affinity maturation. This could be due to differences in immunogenicity or structural complexity of these epitopes. The larger, full-length R21 antigen could trigger a stronger immune response, promoting affinity maturation, whereas smaller epitopes might not generate a robust response or experience comparable affinity enhancements.

There was an overall increase in total cTfh frequency post-challenge, with minimal changes in the Tfh subsets; Th2 and Th17 cells remained the predominant cTfh populations. This pattern mirrors the response observed after vaccination. The presence of antigen-specific MBCs at later time points and their continued presence post-challenge indicate that R21/Matrix-M vaccination may contribute to durable immune memory, potentially enhancing protection against malaria.

Key limitations include the small sample size of some assays, which decreased the statistical power and restricted the generalisability of the results.

Overall, these data highlight the importance of adopting a holistic approach when studying immunity against malaria and vaccine-induced protection. R21/Matrix-M drives multifaceted immunity by combining high antibody titres and isotypes, complement activation, and Tfh-mediated B cell support. While antibody avidity and post-challenge boosts showed limitations, the vaccine’s layered response by activating several key arms of adaptive immunity highlights its potential for durable protection.

## Data Availability

The original contributions presented in the study are included in the article/**Supplementary Material**. Further inquiries can be directed to the corresponding authors.
