# Supplementary material for: R21/Matrix-M malaria vaccine drives diverse immune responses in pre-exposed adults: insights from a phase IIb controlled human malaria infection trial
Source: Front Immunol. 2025 Jul 7;16:1620365. doi: 10.3389/fimmu.2025.1620365 (PMC12277344; doi:10.3389/fimmu.2025.1620365)
Supplement: Supplementary file 1 [file DataSheet1.pdf]

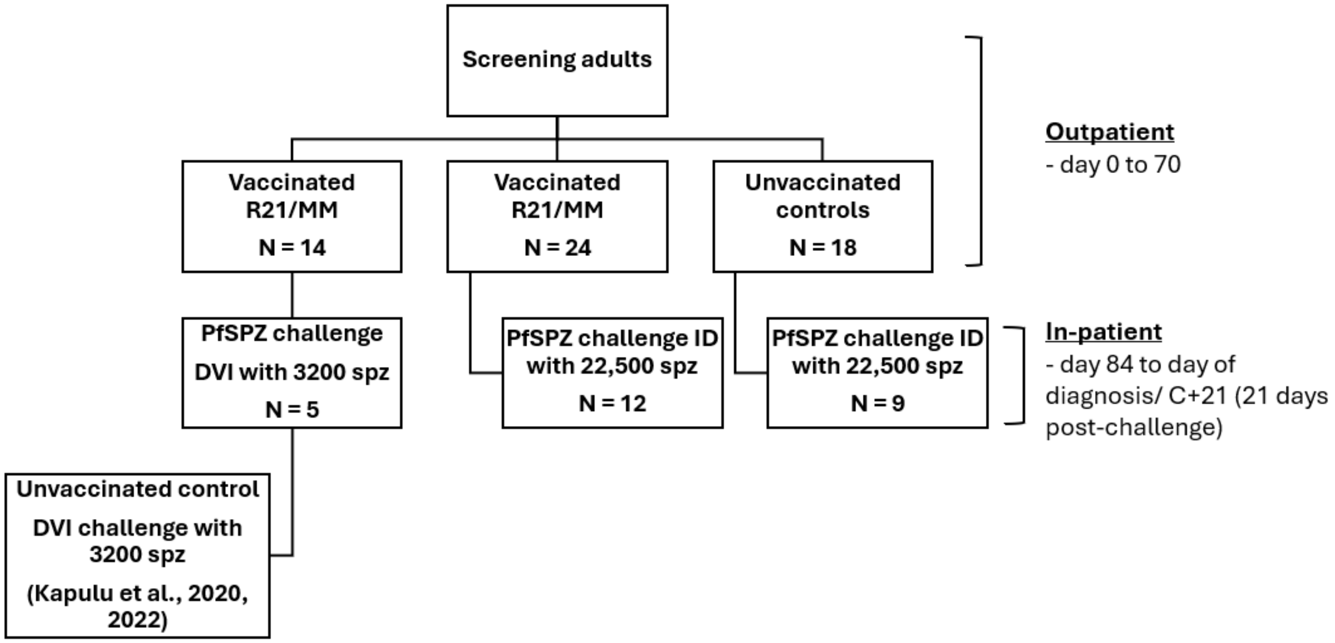

S1: Schematic representation of the VAC 074 Trial Design.
Study participants were adults recruited from Ngerenya in Kilifi County, on the East coast of Kenya, a region with low malaria transmission. Volunteers were randomised into three groups: two received three monthly doses of R21/Matrix-M, and one was a control group. One month after the final dose, all groups were challenged with cryopreserved PfSPZ via either intradermal (ID) injection or direct venous inoculation (DVI). Historical data from a previous study of unvaccinated volunteers from the same location who received 3,200 PfSPZ by DVI were included as controls for vaccinees challenged by DVI.

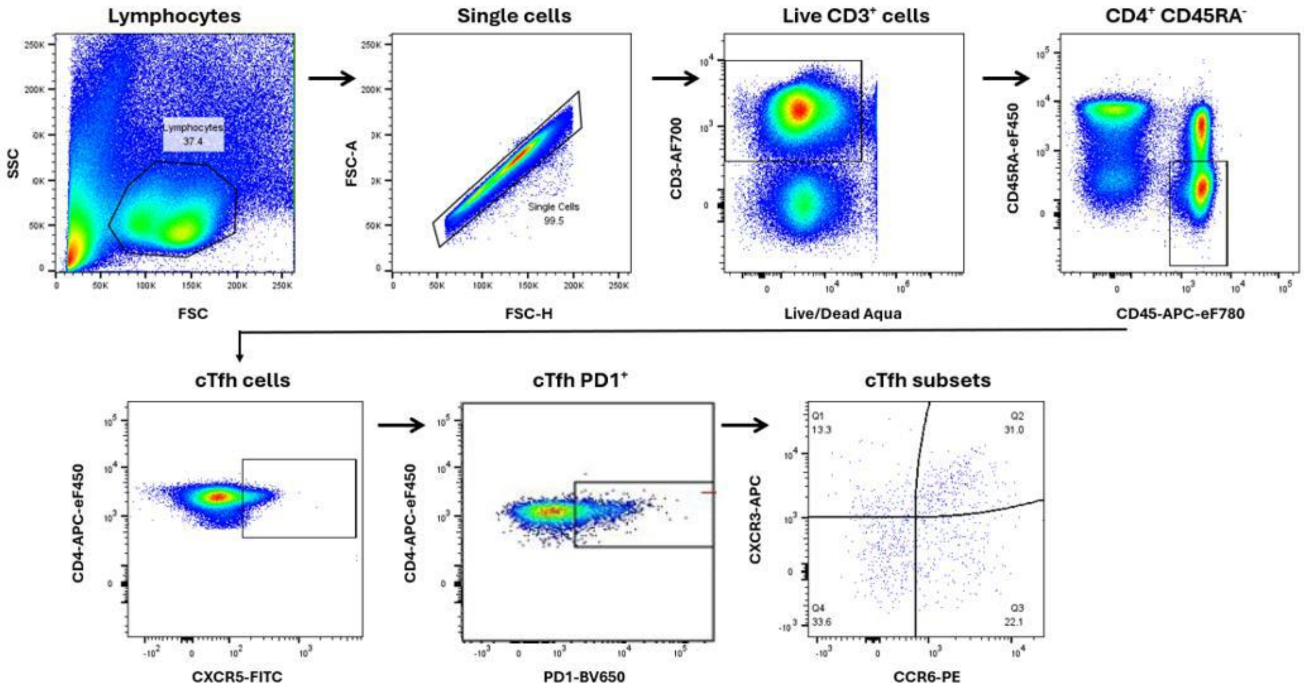

Figure S2: Gating strategy for *ex vivo* cTfh phenotyping
PBMCs were thawed, stained, and analysed by flow cytometry. Lymphocytes were gated based on the forward scatter area (FSC-A) and side scatter area (SSC-A), and single cells were selected using FSC height (FSC-H) versus FSC-A to exclude cell aggregates. T cells were identified as Live/Dead Aqua-negative (indicating viable cells) and CD3-positive. Within the CD4+ T cell population, memory CD4+ T cells were defined as CD45RA-negative, and memory T follicular helper (Tfh) cells were identified by co-expression of PD1 and CXCR5. Further phenotyping of memory CD4+ and Tfh cells was performed using CXCR3 and CCR6 expression levels. Memory Tfh cells were categorized as Tfh1 (CXCR3+ CCR6+), Tfh2 (CXCR3- CCR6-), and Tfh17 (CXCR3- CCR6+).

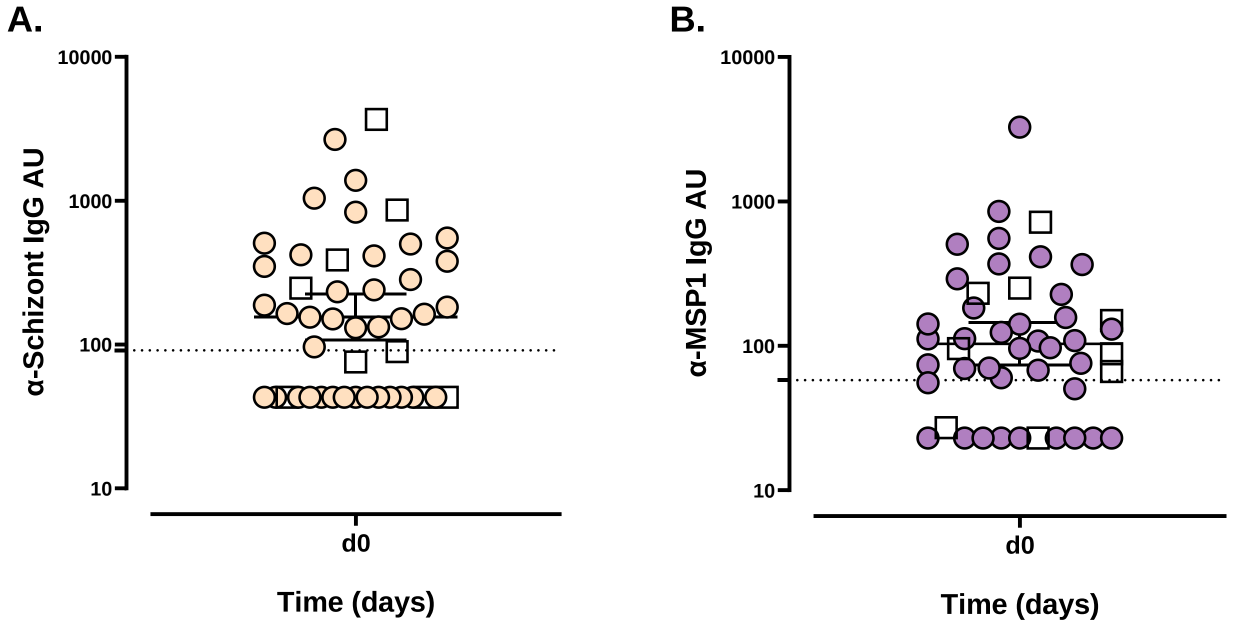

Figure S3: Naturally acquired anti-*Plasmodium* IgG responses.
Anti-*Plasmodium* IgG measured by standardized ELISA at baseline (day 0) to determine the previous malaria exposure in the participants. (A) Schizont ELISA, d0 (*n* = 47); (B) MSP-1 ELISA, d0 (*n* = 47). Results are reported in arbitrary units (AU) and presented as geometric mean titres (GMT) with 95% ±95% CI. The ELISA detection limit was an optical density (OD) of 0.2. Samples with OD <0.2 were assigned a minimum AU value of 43 in the Schizont ELISA and AU 23 in the MSP-1 ELISA. The y-axis is displayed on a log10 scale. The horizontal dashed line indicates the positivity threshold (calculated as the mean plus 3x Standard deviation from U.K naïve individuals). In both graphs, filled circles represent participants who were randomised to receive the R21/Matrix-M vaccination, whereas open squares denote control participants.

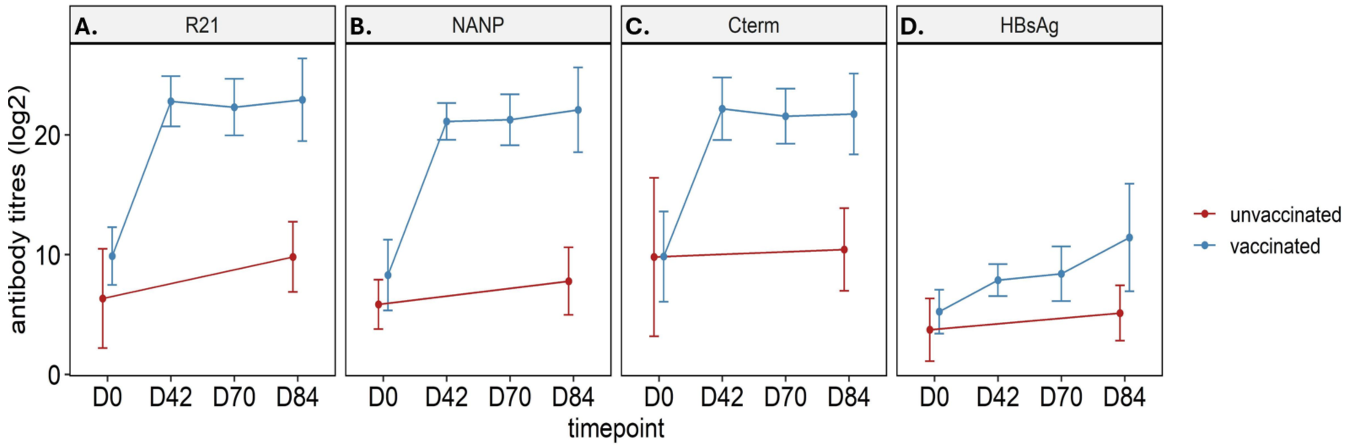

Figure S4: IgG responses measured using the MSD multiplex assay.
IgG antibody responses to the individual components of the R21/Matrix-M vaccine were assessed using the MSD assay two weeks after each vaccination. (A) Response to full-length R21, (B) response to NANP, (C) response to C-terminus, and (D) response to HBsAg. Antibody levels are shown as grouped medians with interquartile ranges (IQR) at each time point.

Supplementary Table 1: Sequence information on CSP proteins

Antigen		Sequence	Manufacturer
R21	Protein	MDPNANPNANPNANPNANPNANPNANPNANPNANPNANPNANPNANPNANP<br>NANPNANPNANPNANPNANPNA<br>NPNANPNKNNQGNGQGHNMPNDPNRNVDENANANSAVKNNNNEEPSD<br>KHIKEYLNKIQNSLSTEWSPCSVTCGNGIQVRIKPGSANKPKDELDYANDIEKKICKMEKCSSVPV<br>TNMENITSGFLGPLLVLQAGFFLLTRILTIPQSLDSWWTSLNFLGGSPVCLGQNSQSPTSNHSP<br>TSCPPICPGYRWMCLRRFIIFLFILLLCLIFLLVLLDYQGMLPVC<br>PLIPGSTTTNTGPCKTCTTPAQGNSMFPSCCCTKPTDGNCTCIPIPSSW<br>AFAKYLWEWASVRFSWLSLLVPFVQWFVGLSPTVWLSA<br>IWMMWYWGPSLYSIVSPFIPLLPIFFC LWVYI	Serum Institute of India
NANP6	Peptide	NANPNANPNANPNANPNANPNANP	Thinkpeptides, Proimmune, Oxford, UK
C-terminus	Peptide	epsdkhikeylnkiqnslstewspcsvtcgngiqvrikpgsankpkdeldyandiekkickmekcs	Thinkpeptides, Proimmune, Oxford, UK

Supplementary Table 2: Staining antibody panel of the identification of total Tfh and Tfh sub-sets

Marker	Fluorophore	Manufacturer	Clone	Dilution
**CD3**	AF700	ebiosciences	UCHT1	1:33
**CD4**	APC-eF780	ebiosciences	RPA-T4	1:50
**CD45RA**	eF450	Biolegend	HI100	1:50
**PD-1**	BV650	Biolegend	EH12.2H7	1:100
**CXCR5**	FITC	Biolegend	J252D4	1:50
**CCR6**	PE	Biolegend	G034E3	1:25
**CXCR3**	APC	Biolegend	G025H7	1:17
**Live/dead**	Aqua	Life Technologies	n/a	1:500

Supplementary Table 3: Baseline demographic characteristics [13].

Characteristic	R21 (Intradermal CHMI) n = 24	R21 (Intravenous CHMI) n = 14	Control (Intradermal CHMI) n =18	Overall n = 80
Age in years, mean (SD)	28 (6)	26 (5)	29 (6)	28 (6)
Sex, female, n (%)	4 (17%)	4 (29%)	6 (33%)	23 (29%)
Sex, male, n (%)	20 (83%)	10 (71%)	12 (67%)	57 (71%)
BMI, kg/m^2, mean (SD)	21.7 (2.0)	20.2 (1.8)	20.2 (1.7)	21.7 (3.1)
PCR (parasites), median [min-max]	0 [0-0]	0 [0-0.0167]	0 [0-0]	0 [0-0.0167]
PCR positive, n (%)	0 (0%)	1 (7.1%)	0 (0%)	1 (7.1%)
n = number of healthy volunteers enrolled to each vaccination group. BMI, Body mass index, PCR (parasites/µl) was recorded at pre-vaccination (pre-vac). Data are presented as either mean and SD (standard deviation), medium [min-max] for continuous variables and n (%) for categorical variables. Percentages are presented with denominator as number of healthy volunteers per vaccination groups.				
